# Lysyl Oxidase Reduces Neutrophil Extravasation in Response to *P. aeruginosa* in an Infection‐on‐a‐Chip Model

**DOI:** 10.1002/anbr.202500180

**Published:** 2026-03-07

**Authors:** Christopher J. Calo, Tanvi Patil, Josephine Fouts, Eric L. Ginter, Margaret Radke, Hannah K. Weppner, Justin M. Owens, Laurel E. Hind

**Affiliations:** ^1^ Chemical and Biological Engineering University of Colorado – Boulder Boulder Colorado USA

**Keywords:** extravasation, infection‐on‐a‐chip, lysyl oxidase, neutrophil, transendothelial migration, vascular endothelial cadherin

## Abstract

Neutrophils are crucial players in the fight against infections. Unfortunately, dysregulated neutrophil function contributes to the pathogenesis of diseases, including cancer, fibrosis, and atherosclerosis, that leave those afflicted vulnerable to severe infections. Many of these diseases are accompanied by differential lysyl oxidase (LOX) activity. The LOX enzyme crosslinks collagen and elastin, two highly expressed proteins in the extracellular matrix (ECM), altering the ECM's mechanical properties. While it is known ECM mechanical properties regulate neutrophil function, the role of LOX crosslinking of collagen on the neutrophil response is unclear. This study uses a microfluidic “infection‐on‐a‐chip”. This device consists of a model blood vessel endothelium embedded in an ECM mimic to investigate how LOX crosslinking of collagen affects neutrophil function in response to infection. Interestingly, LOX‐crosslinking of collagen decreases neutrophil extravasation through an endothelium in response to *Pseudomonas aeruginosa* compared to uncrosslinked collagen hydrogels. Critically, endothelial cells in devices with LOX‐crosslinked collagen exhibited increased VE‐cadherin expression compared to those seeded in uncrosslinked collagen gels, which is hypothesized to restrict neutrophil extravasation. These data demonstrate the regulatory capability of LOX over the neutrophil response, providing a potential therapeutic pathway for diseases associated with neutrophil dysregulation and LOX activity that merits further investigation.

## Graphical abstract

Lysyl oxidase crosslinking of type I collagen decreases neutrophil extravasation through an endothelium during the initial response to *P. aeruginosa*. This decrease in extravasation is likely due to the increase in VE‐cadherin expression between endothelial cells seeded on LOX‐crosslinked collagen hydrogels.

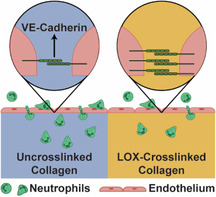

## 1 Introduction

Neutrophils champion the early innate immune response to infections. Proper regulation of neutrophils is crucial as overactivation leads to chronic inflammation and damage to otherwise healthy host tissue, while an underactive response allows for severe and recurrent infections. Indeed, dysregulated neutrophil function has been identified as contributing to the pathogenesis of diseases and conditions, such as fibrosis, cancer, and atherosclerosis [1, 2, 3, 4, 5]. These conditions leave patients vulnerable to infection. Current efforts have identified the neutrophils as a therapeutic target to combat such diseases and infections [6, 7, 8, 9, 10]. Effective design of such therapeutics requires a thorough understanding of the factors that govern the neutrophil response; however, our understanding of these regulatory factors is incomplete. Interestingly, diseases associated with neutrophil dysregulation are often accompanied by alterations to the composition of the surrounding extracellular matrix (ECM) [11, 12, 13, 14]. Notably, differential expression of lysyl oxidase (LOX), a collagen and elastin crosslinking enzyme, has been implicated in the disease progression of atherosclerosis, fibrosis, and cancer [15, 16, 17, 18].

The lysyl oxidase family of enzymes oxidize lysyl and hydroxylysyl residues of collagen and elastin in a copper‐dependent manner to facilitate covalent crosslinking of the ECM. This crosslinking is a crucial step for matrix stabilization and mechanical integrity [19]. Studies using tunable hydrogels with varying mechanical properties have explored the mechanosensitive nature of neutrophils [20, 21, 22, 23, 24, 25, 26, 27, 28]. Neutrophil extravasation through an endothelial monolayer increases with increasing stiffness of the underlying [25, 29]. Additionally, mouse neutrophils encapsulated in stiff GelMA hydrogels have increased chemotaxis toward CXCL8 compared to those encapsulated in soft hydrogels [30]. Thus, it is possible that LOX crosslinking of the ECM could significantly impact neutrophil extravasation and migration.

While neutrophils drive the early innate immune response, their activation is heavily reliant on the endothelial cells that line blood vessels near the inflammatory site [31, 32]. As with neutrophils, endothelial cells are mechanosensitive [29, 33, 34, 35, 36, 37] and endothelial stiffness is directly correlated with the stiffness of the underlying substrate [35]. Endothelial stiffening increases vascular permeability by elongating intercellular junctions and can change the secretion of inflammatory cytokines [38, 39]. LOX affects endothelial cells directly; yet, how this might affect the neutrophil response is unclear. LOX is highly expressed in healthy coronary arteries, and its downregulation leads to endothelial dysfunction associated with atherosclerosis [40]. In tumor environments, LOX promotes angiogenesis of endothelial cells [41, 42]. Interestingly, in fibrosis, upregulated LOX activity has been linked to transforming growth factor β1 signaling, a potent neutrophil chemoattractant produced by endothelial cells [43, 44, 45]. Given all the avenues through which LOX directly and indirectly affects endothelial cell behavior and their role in the neutrophil response, when studying LOX regulation of the neutrophil response it is important to use a system that includes an endothelium.

In this study, we investigated the effects of LOX crosslinking on the neutrophil response to *Pseudomonas aeruginosa*, an opportunistic gram‐negative bacterial pathogen that poses significant risk to immunocompromised patients, such as those with altered LOX crosslinking. We carried out these studies using our “infection‐on‐a‐chip” microfluidic device, which includes a model blood vessel embedded in a collagen matrix [46, 47, 48]. We determined that LOX crosslinking of collagen decreased neutrophil extravasation in response to *P. aeruginosa*, likely due to increased expression of vascular endothelium cadherin (VE‐cadherin) in the junctions between endothelial cells. However, it did not alter the migration patterns of extravasated neutrophils. These findings highlight the importance of understanding ECM‐derived regulators of the neutrophil response, indicating a role for LOX regulation of neutrophil extravasation.

## 2 Results

### 2.1 Crosslinking Collagen With Lysyl Oxidase Marginally Increases Modulus But Does Not Affect Matrix Pore Size

**FIGURE 1:**
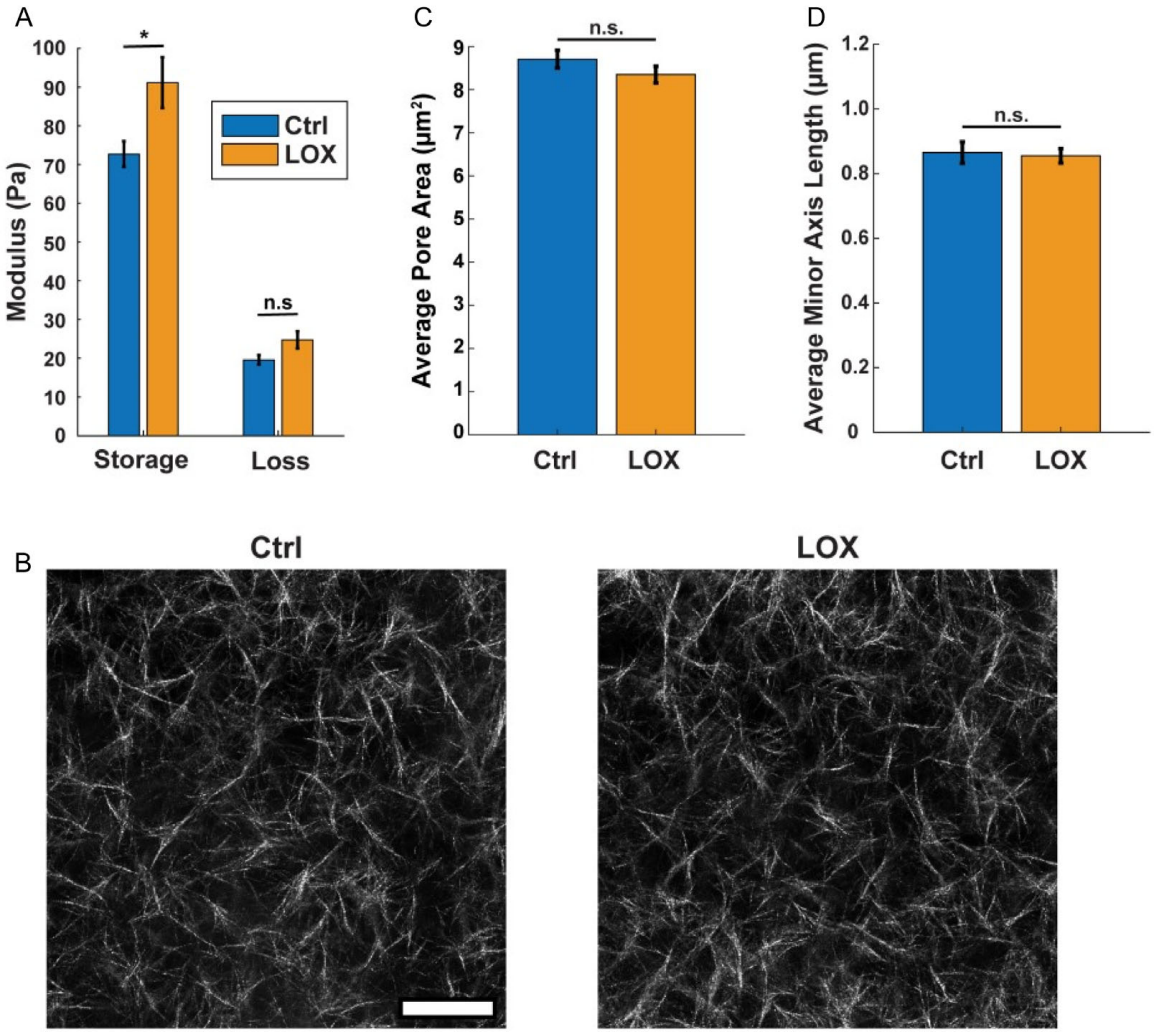
Crosslinking collagen with lysyl oxidase moderately increases the storage moduli of type I collagen gels. (A) Storage (G′) and Loss (G″) moduli of LOX‐crosslinked (LOX) and uncrosslinked (Ctrl) hydrogels after 5 days of treatment, measured at 10 rad s^−1^, 1.0% strain measured at 37°C. Error bars indicate mean ± SEM of 16 gels per condition across 3 independent experiments. Student's *t*‐test was performed to compare the two conditions with an alpha value of 0.05. Asterisks signify where *p* < 0.05. (B) Representative confocal reflectance images of LOX‐crosslinked (LOX) and uncrosslinked (Ctrl) gels (scale bar = 50 μm). Average (C) area and (D) minor axis length (M.A.L.) of pores for both conditions (Ctrl, 49 994 pores and LOX, 51 556 pores; across 12 different gels per condition). Dunn–Sidak tests were performed to compare the pore properties between the Ctrl and LOX conditions with an alpha value of 0.05. Error bars specify mean ± SEM.

Lysyl oxidase (LOX) is a copper‐dependent enzyme that crosslinks proteins in the ECM by oxidizing lysyl and hydroxylysyl residues, such as those found in collagen peptides. Incorporating covalent crosslinks into a gel system can change the stiffness and pore size of the network. Thus, it was important to characterize these features of the collagen gels used in this study. 4 mg mL^−1^ type I collagen disks were soaked in endothelial growth medium (EGM‐2) supplemented with 10% fetal bovine serum and either no LOX or 150 ng mL^−1^ LOX for 5 days. This treatment scheme has previously been shown by the Erler Group to increase the moduli of type I collagen gels comparably to glycating gels with ribose, a known method for increasing collagen stiffness [17, 49]. Then, oscillatory rheology was performed to determine the Storage and Loss Moduli of the gels (Figures 1A and S1A). The LOX‐crosslinked gels (LOX) showed a statistically significant 1.25‐fold increase in Storage Modulus compared to the uncrosslinked gels (Ctrl), which agrees with previously reported values [17, 49].

We next used confocal reflectance microscopy to quantify the effect of LOX crosslinking on pore size and distribution in the collagen gels (Figure 1B). The mean values and distributions of pore area and minor axis length (M.A.L.) were determined using Matlab's bwlabel and regionprops functions (Figures 1C,D and S2). The average pore area and M.A.L. of uncrosslinked (Ctrl) gels were 8.70 ± 0.21 μm^2^ and 0.865 ± 0.033 μm, respectively. By comparison, the average pore area and M.A.L. of the LOX‐crosslinked (LOX) gels were 8.35 ± 0.19 μm^2^ and 0.855 ± 0.022 μm, respectively. There was no significant difference in the pore area or M.A.L. between the two conditions. Together, these data show that treating type I collagen gels with LOX for 5 days marginally increases the Storage Modulus, without affecting the pore structure of the gels.

### 2.2 LOX Crosslinked Collagen Decreases Neutrophil Extravasation to *P. aeruginosa*

**FIGURE 2:**
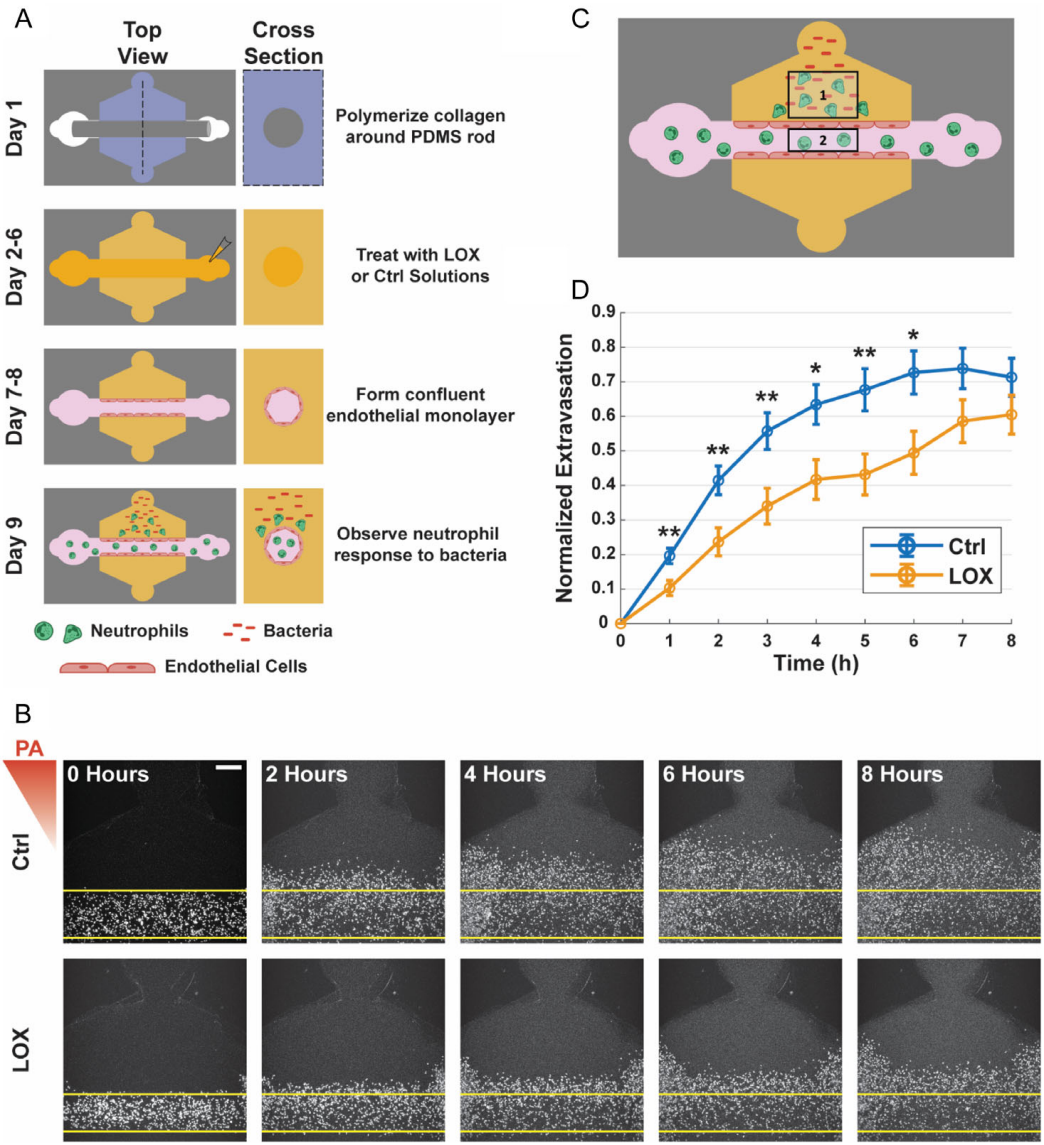
Lysyl oxidase crosslinking of collagen initially decreases neutrophil extravasation in response to *P. aeruginosa*. (A) Schematic representation of how the infection on a chip model is set up and run to observe neutrophil response to bacterial challenges. (B) Representative images of extravasated neutrophils in LOX‐crosslinked (LOX) and uncrosslinked (Ctrl) collagen gels every 2 h for the first 8 h after stimulation with *P. aeruginosa* (scale bar = 250 μm). Yellow lines indicate the top and bottom edges of the lumen. The red triangle shows the initial *P. aeruginosa* (PA) concentration gradient. (C) Illustration of the analysis protocol for determining normalized extravasation. (D) Normalized number of extravasated neutrophils into LOX and Ctrl collagen gels. Data quantified from 20 Ctrl devices  and 19 LOX devices across 4 independent experiments and 4 neutrophil donors. For each timepoint, Student's *t*‐test was used to compare the extent of extravasation between Ctrl and LOX conditions with an alpha value of 0.05 followed by Tukey‐Kramer adjustment. Error bars indicate the means ± SEM. Asterisks indicate significance conditions at the same timepoint where *p < 0.05 and **p < 0.01.

Once the effects of LOX on type I collagen were characterized, we investigated how LOX‐crosslinked collagen affects neutrophil extravasation, a crucial step in the neutrophil response to infection. To do this, we used our “infection‐on‐a‐chip” device that includes a portion of a blood vessel endothelium embedded in a natural ECM hydrogel, which can be used to study the human neutrophil response to infection [48]. For these studies, type I collagen was treated with EGM‐2 supplement with 10% FBS and either no LOX (Ctrl solution) or 150 mg mL^−1^ LOX (LOX solution) for 5 days post collagen polymerization in the device. Then, human umbilical vein endothelial cells (HUVECs) were seeded into the side ports and formed a confluent monolayer over 2 days. For extravasation experiments, primary human neutrophils were stained, seeded into lumens, and extravasation was induced with *P. aeruginosa* (Figure 2A). Neutrophil extravasation was imaged using confocal microscopy over an 8‐h period following stimulation with *P. aeruginosa* (Figure 2B). Importantly, we found that LOX crosslinking had no effect on *P. aeruginosa* distribution throughout the device (Figure S3). Extravasated neutrophils were quantified as described previously [46]. Briefly, normalized values for extravasated neutrophils were determined by counting the number of neutrophils in a region of interest (ROI) immediately above the top edge of the endothelial lumen and dividing by the number of neutrophils in a second ROI inside the lumen at *t* = 0 (Figure 2C). Significantly fewer neutrophils extravasated into the LOX crosslinked collagen compared to the untreated collagen during the first 6 h post *P. aeruginosa* stimulation (Figure 2D). This indicates that the LOX activity reduces the initial burst of neutrophils extravasating in response to *P. aeruginosa*.

### 2.3 Neutrophil Motility is Unaffected by LOX Crosslinking of Collagen

**FIGURE 3:**
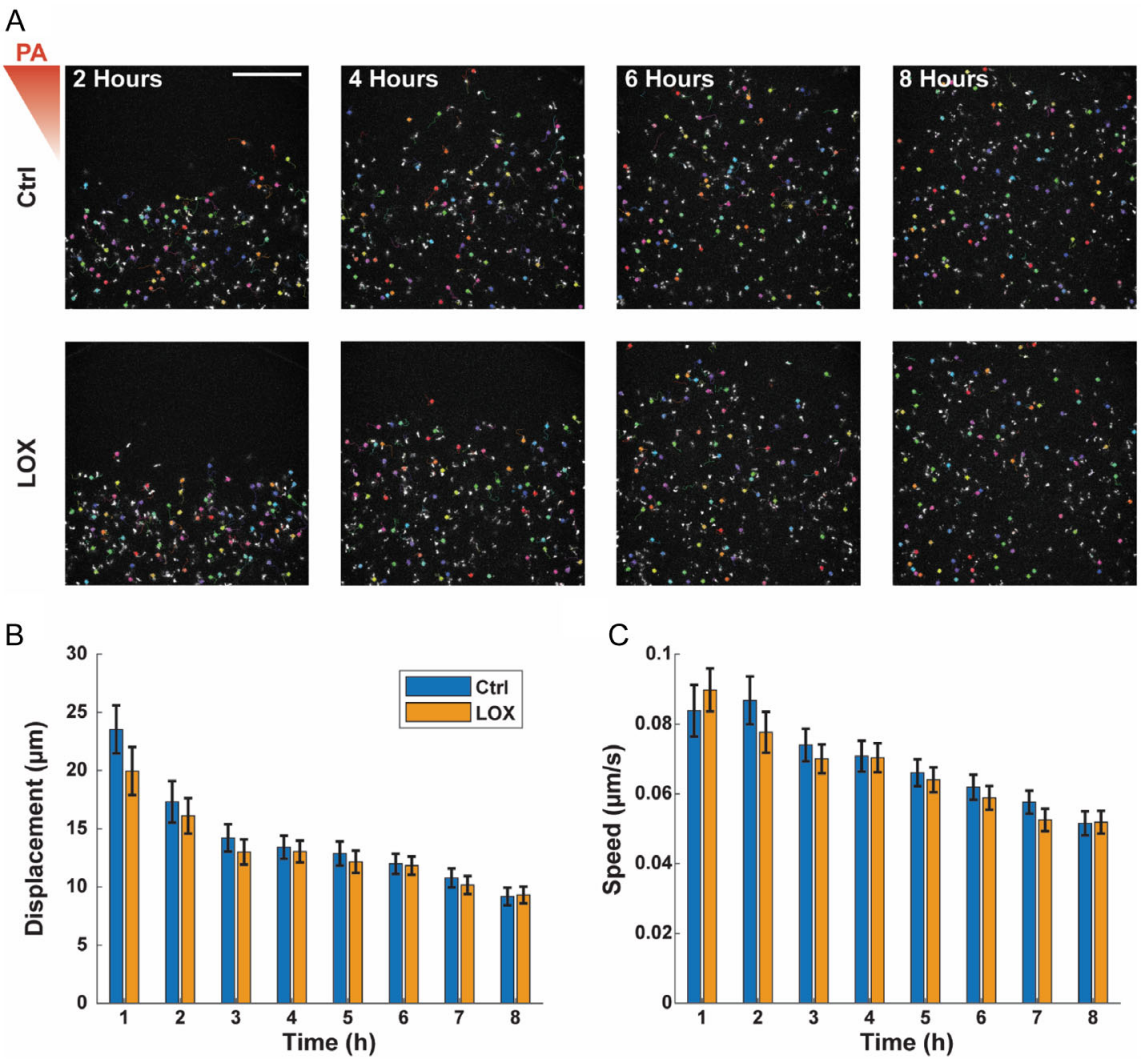
Migration patterns of extravasated neutrophils are not affected by lysyl oxidase crosslinking of collagen. (A) Representative images and tracks of migrating neutrophils in LOX‐crosslinked (LOX) and uncrosslinked (Ctrl) collagen gels every 1 h for the first 8 h after stimulation with *P. aeruginosa* (scale bar = 250 μm). Each individual track is shown in different colors. The red triangle shows the initial *P. aeruginosa* (PA) concentration gradient. Migration properties: (B) displacement and (C) speed of neutrophils quantified over 10‐min increments every hour for 8 h after stimulation with *P. aeruginosa*. Extravasated neutrophils, from 20 Ctrl devices and 19 LOX devices across 4 independent experiments and 4 neutrophil donors, were tracked using the cell motility function in Nikon's Elements Software. LOX‐crosslinked and uncrosslinked conditions were compared to each other at each time point using Student's *t*‐test with an alpha value of 0.05 followed by a Tukey‐Kramer adjustment. Error bars indicate the means ± SEM.

After extravasating, neutrophils maneuver through the ECM to reach the site of infection. To investigate how LOX crosslinking of collagen affects neutrophil motility in the presence of *P. aeruginosa*, we quantified migration parameters of extravasated neutrophils in the untreated and LOX‐crosslinked collagen gels in our infection‐on‐a‐chip device. Extravasated neutrophils were imaged every 30 s for 10‐min intervals every hour for 8 h after *P. aeruginosa* stimulation (Figure 3A). Migration properties were calculated as has been described previously [46]. Briefly, net displacement was calculated as the distance between the beginning and ending positions for each 10‐min imaging period (Figure 3B). The straightness of migration was calculated as the migration path length divided by the displacement to determine the straightness of migration (Figure S4). Finally, speed was computed as the total path length divided by the time elapsed during the cell's tracked migration (Figure 3C). There were no significant differences between the Ctrl and LOX conditions for neutrophil migration displacement, speed, path length, or straightness. Together, these data show no significant differences in neutrophil migration patterns over 10‐min intervals during the 8 h poststimulation with *P. aeruginosa.*

### 2.4 Collagen Crosslinking by LOX Does Not Significantly Alter Barrier Function

**FIGURE 4:**
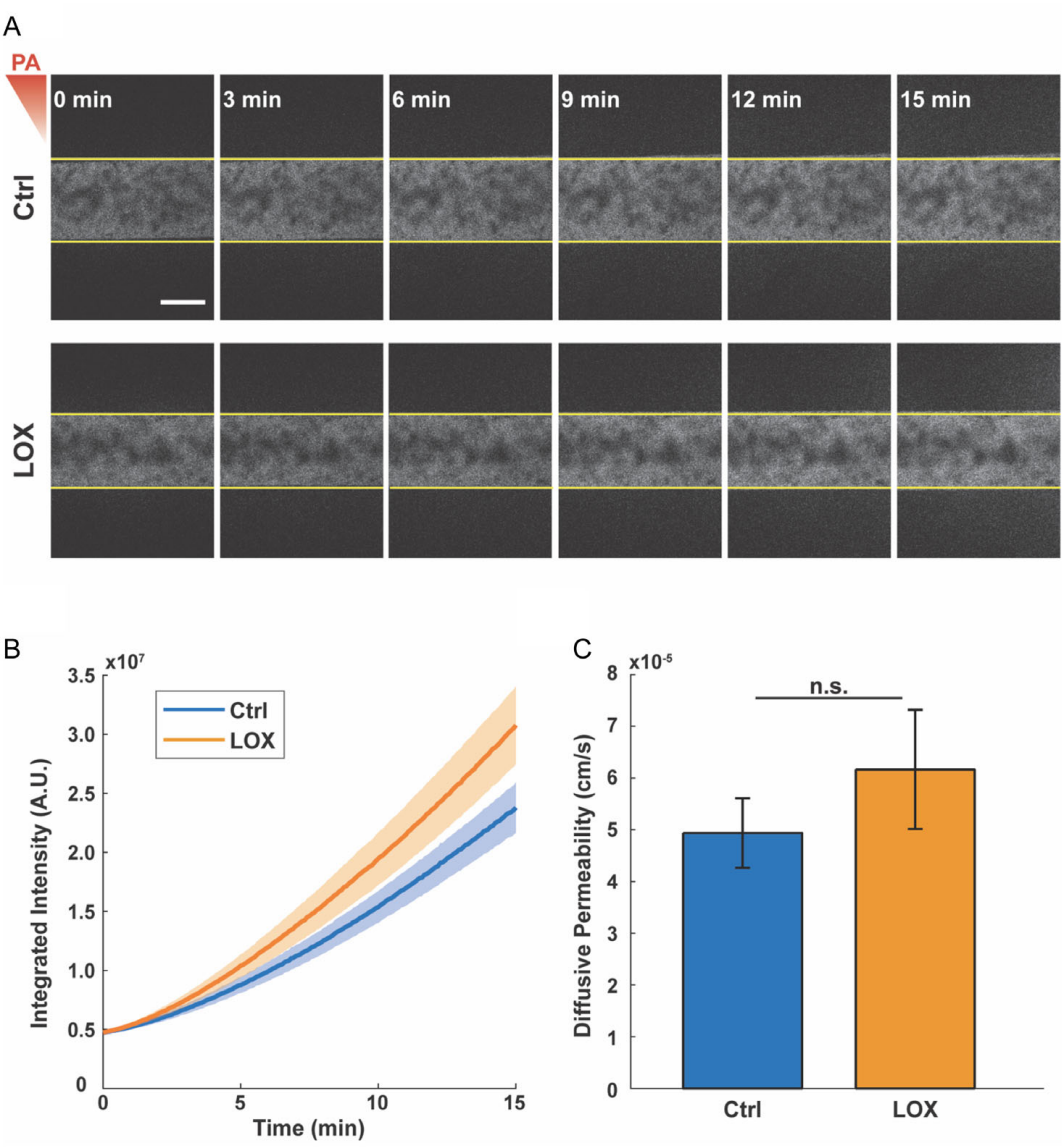
LOX crosslinking of collagen does not affect endothelial diffusive permeability. (A) Representative images of 10 kDa fluorescein isothiocyanate‐dextran diffusion out of endothelial lumens into LOX‐crosslinked (LOX) and uncrosslinked (Ctrl) collagen gels at 0, 3, 6, 9, 12, and 15 min, 2 h post stimulation with *P. aeruginosa* (scale bar = 250 μm). Yellow lines indicate the top and bottom edges of the lumen. The red triangle shows the initial *P. aeruginosa* (PA) concentration gradient. (B) Plot of integrated intensity in the gel above the top lumen edge over time. Lines indicate the mean intensity from 14 devices for both the Ctrl and LOX conditions across 3 independent experiments and the shaded regions show the mean ± SEM. (C) Diffusive permeability of 10 kDa dextran into LOX and Ctrl collagen gels. Student's *t*‐test with an alpha value of 0.05 was used to compare the two conditions. Error bars indicate the means ± SEM.

We next sought to understand how crosslinking collagen with LOX reduced neutrophil extravasation. The presence of an endothelium has previously been shown to be crucial for proper neutrophil extravasation in response to inflammatory stimuli [46, 48]. However, the endothelium also serves as a barrier, preventing blood from leaking into the surrounding ECM. Previous works have shown that LOX activity impacts endothelial barrier function; therefore, we quantified how crosslinking collagen with LOX affected the diffusive permeability of the endothelia in the microfluidic system [40, 41, 42, 50, 51]. Devices were prepared as before; however, no neutrophils were added to the lumens. Instead, after the devices had incubated with *P. aeruginosa* for 2 h, 10 kDa dextran functionalized with fluorescein isothiocyanate in EGM‐2 was added to the lumens and the diffusion of dextran into the surrounding hydrogel was observed via confocal microscopy every 5 s for 15 min (Figure 4A). The integrated fluorescence intensity in the hydrogel above the top lumen edge was plotted over time to visualize dextran accumulation (Figure 4B). Then, the diffusive permeability was calculated using the slopes from the lines of best fit for the first 5 min of the integrated intensity plots from Figure 4B following the method previously described by Polacheck et al. (Figure 4C) [52]. We found no significant differences in diffusive permeability, suggesting that LOX crosslinking of collagen did not affect vascular barrier function.

### 2.5 LOX Crosslinking of Collagen Results in Increased VE‐Cadherin Expression between Endothelial Cells during Infection with *P. aeruginosa*

**FIGURE 5:**
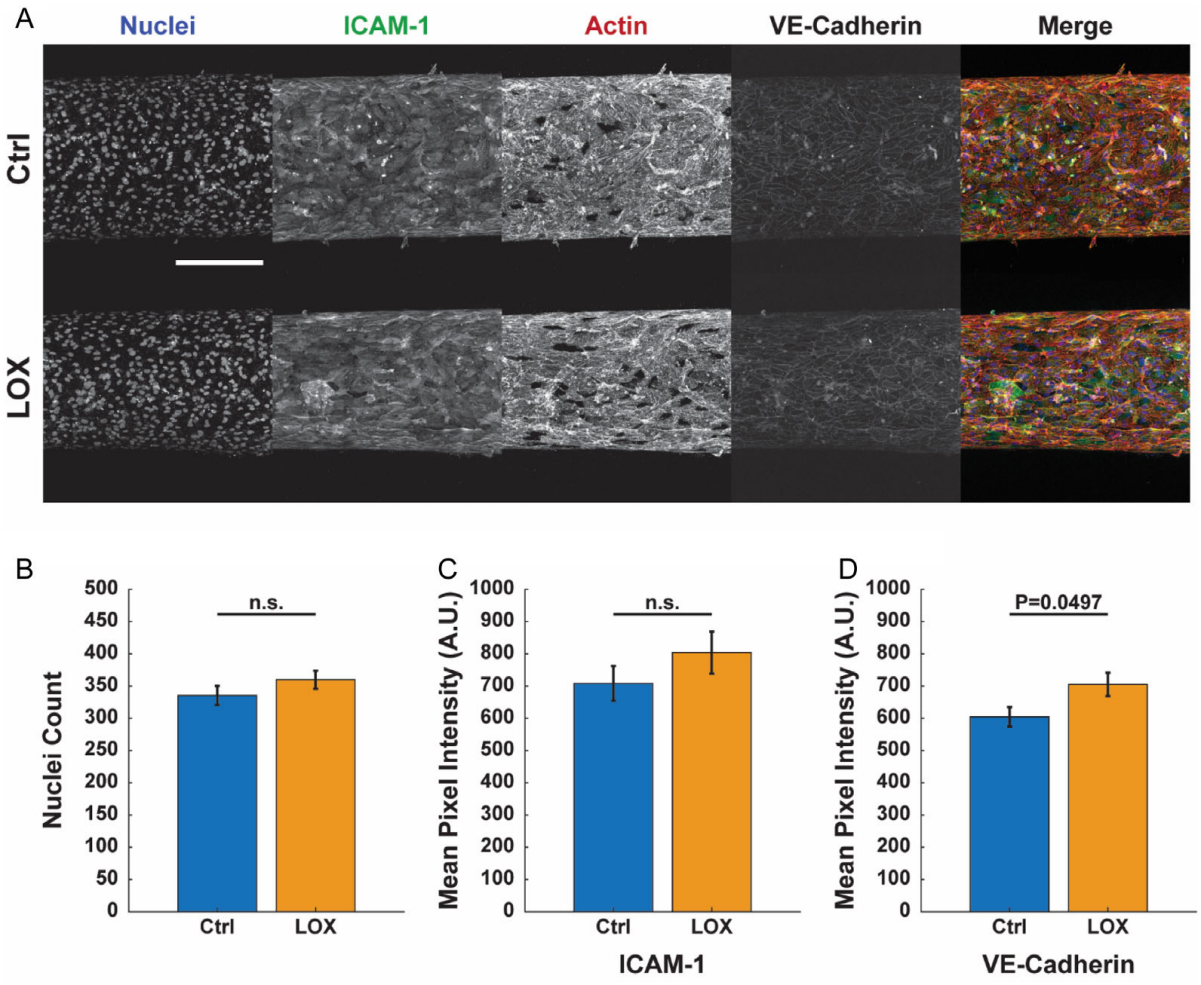
Lysyl oxidase crosslinking of collagen increases VE‐cadherin between endothelial junctions but not surface ICAM‐1 expression. (A) Representative maximum intensity projections of the bottom half of HUVEC lumens seeded in microfluidic devices surrounded by LOX‐crosslinked (LOX) or uncrosslinked (Ctrl) collagen gels and stained with Hoechst (nuclei, blue), anti‐ICAM‐1 (adhesion molecules, green), phalloidin (actin, red), anti‐VE‐cadherin (junctions, far red) 2 h post stimulation with *P. aeruginosa* (scale bar = 250 μm). Bar graphs of (B) the number of nuclei counted, (C) mean pixel intensity of ICAM‐1, and (D) mean pixel intensity of VE‐cadherin in a fixed region of interest about the bottom of each lumen. Nuclei counts and mean pixel intensities from 9 endothelial lumens per condition across 3 independent experiments were compared to each other via Student's *t*‐test with an alpha value of 0.05 followed by a Tukey‐Kramer adjustment. Error bars indicate mean ± SEM.

Endothelial cell surface protein expression of adhesion molecules and junctional proteins regulate neutrophil activation and extravasation [32, 53, 54]. Additionally, LOX activity has been implicated in regulating expression of adhesion molecules, including intercellular adhesion molecule 1 (ICAM‐1), as well as vascular permeability [50, 51, 55]. Therefore, we sought to quantify the expression of adhesion and junctional proteins, namely ICAM‐1 and VE‐cadherin, respectively, by the HUVECs in Ctrl and LOX gels in the microfluidic system. *P. aeruginosa* was added to the top ports of LOX‐crosslinked (LOX) and uncrosslinked (Ctrl) microfluidic systems and incubated for 2 h prior to fixing and staining to visualize endothelial nuclei (Hoechst), adhesion molecules (anti‐ICAM‐1), actin (phalloidin), and junctions (anti‐VE‐cadherin) with confocal microscopy (Figure 5A). There were no significant differences in the number of endothelial nuclei or ICAM‐1 intensity between the two conditions (Figure 5B,C). However, VE‐cadherin intensity was significantly higher in the LOX condition compared to the Ctrl condition (Figure 5D). These trends were also seen in endothelial cells plated in a well plate (Figure S5). These data suggest LOX crosslinking of collagen increases junctional VE‐cadherin between endothelial cells, which may be responsible for the observed decrease in neutrophil extravasation in the LOX condition compared to the Ctrl condition.

## 3 Conclusion

In this study, we investigated the effects of enzymatically crosslinking collagen on the neutrophil response to *P. aeruginosa* in a physiologically relevant “infection‐on‐a‐chip” device. Our data showed less neutrophil extravasation during the first 6 h in response to *P. aeruginosa* into LOX‐crosslinked collagen gels. Unlike extravasation, there were no significant differences in the migration patterns of extravasated neutrophils during any of the observed 10‐min intervals. Additionally, in the presence of *P. aeruginosa*, in the LOX‐crosslinked gels we observed increased VE‐cadherin expression compared to uncrosslinked gels in our device*.* These results suggest that LOX activity may decrease the initial extravasation step of the neutrophil infectious response by increasing junction integrity between endothelial cells.

Neutrophils are first responders during a bacterial infection, often responding within minutes to hours of an initial infection [56, 57]. This response is critical as defects in neutrophil accumulation in the tissue can lead to serious or recurrent infections. We found that neutrophil extravasation was significantly reduced in LOX‐crosslinked gels for the first 6 h following infection with *P. aeruginosa* in our microfluidic device. This could have significant implications for early control of infection. Our study focused on the neutrophil response to *P. aeruginosa*, an opportunistic gram‐negative pathogen associated with underlying conditions such as those associated with increased LOX [58, 59]. However, we have previously reported that the magnitude of the neutrophil response varies in response to differing bacterial pathogens [47]; therefore, the effect of LOX‐crosslinking on the neutrophil response may be pathogen dependent and future studies should explore this possibility.

LOX is a copper‐dependent enzyme that stiffens the ECM by crosslinking fibrous proteins such as collagen and elastin [19]. LOX activity changes in diseases associated with neutrophil dysfunction, including atherosclerosis [5, 18, 40]. While it is known that neutrophils are mechanosensitive cells, it is unclear how changing LOX activity affects the neutrophil response. We found greater neutrophil extravasation into the Ctrl gels compared to the LOX gels over the first 6 h post stimulation with *P. aeruginosa*. LOX has previously been shown to increase the stiffness of collagen gels *in vitro* [17, 49, 60] and neutrophil extravasation through HUVECs increases when the underlying matrix stiffness increases [25]. Thus, LOX‐crosslinking of collagen would be expected to increase neutrophil extravasation, yet we observed the opposite. This may be, in part, due to the difference in the magnitude of the changes to stiffness among these studies. *In vitro* LOX‐crosslinking of type I collagen gels generates a marginal increase in stiffness of soft (<1 kPa) gels, whereas the study showing increased neutrophil extravasation with increased matrix stiffness spanned multiple orders of magnitude (0.42–280 kPa), all stiffer than the gels used in this study. Therefore, the stiffening of collagen by LOX in this study may not have been significant enough to induce the previously reported increased extravasation of neutrophils. However, this does not explain the observed decrease in neutrophil extravasation.

Given the importance of the endothelium in neutrophil activation and extravasation, we investigated endothelial surface protein expression in our system to determine if that could explain the observed decrease in neutrophil extravasation. LOX has been shown to alter endothelial cell protein expression. Notably, LOX activity increases ICAM‐1 expression by endothelial cells, which is thought to be the result of LOX stiffening of the subendothelial matrix [55]. ICAM‐1 is crucial for neutrophil adhesion to the endothelium and facilitates extravasation into the ECM [54]. Additionally, multiple studies have shown that decreasing LOX activity with low‐density lipoproteins, β‐aminopropionitrile, or gene knockdown in mice increases vascular permeability [50, 51]. VE‐cadherin is a prominent endothelial junctional protein, whose presence has been implicated in restricting vascular permeability and neutrophil extravasation, though to our knowledge VE‐cadherin expression has not been shown to be directly regulated by LOX activity [39, 53, 61, 62]. Therefore, we quantified the expression of ICAM‐1 and VE‐cadherin during a response to *P. aeruginosa* in endothelial lumens seeded in uncrosslinked and LOX‐crosslinked collagen hydrogels. We found no significant differences in ICAM‐1 expression between the conditions, which may be attributed to the minimal change in matrix stiffness. However, there was significantly more VE‐cadherin expression in the LOX condition compared to the Ctrl. Given VE‐cadherin's role in restricting extravasation, we hypothesize that this increased expression of VE‐cadherin is the reason for the decreased neutrophil extravasation in the LOX condition. Specifically, this would be due to a change in paracellular extravasation, the route of extravasation used by most neutrophils; however, we cannot rule out the possibility that transcellular is altered in the system, which would not rely on VE‐cadherin expression. Interestingly, the early stage of atherosclerosis is characterized by downregulation of LOX activity and increased neutrophil extravasation, in alignment with our results [40, 63]. Thus, it may be the case that the upregulation of neutrophil extravasation can be attributed to decreased VE‐cadherin expression and barrier integrity in the absence of LOX.

Lastly, there were no significant differences in the migration profiles of extravasated neutrophils between the two conditions over the 8 h post stimulation with bacteria. In a 3D environment, neutrophils use amoeboid‐like migration [64, 65]; therefore, the migration profiles of the neutrophils are largely dependent on the pore size of the matrix through which they are migrating [46, 66]. Given that LOX‐crosslinking of the collagen fibrils does not significantly affect the pore size of the gels, it is unsurprising that there were no significant differences in neutrophil motility between the Ctrl and LOX conditions.

Together, these data show that LOX downregulates neutrophil extravasation in response to *P. aeruginosa* in our microfluidic device, likely due to an increase in junctional protein expression between endothelial cells. This is an interesting regulatory mechanism that may be exploited for future therapeutic treatments of diseases associated with LOX dysregulation and neutrophil dysfunction, such as in atherosclerosis. While type I collagen is the predominant ECM protein, it is by no means the only fibrous protein in the ECM. Additional proteins, such as other collagens and elastin, are also present and capable of being crosslinked by LOX. Incorporating these proteins into future experiments may allow for greater matrix stiffening, which may bring about proinflammatory signals that would compete with the anti‐inflammatory signals reported herein, and merits further investigation.

## 4 Materials and Methods

### 4.1 Collagen Preparation

Collagen gels were formed as described previously [46]. Briefly, high concentration type‐I collagen (354249, Corning, Corning, NY) was diluted to a final concentration of 4 mg mL^−1^ by neutralizing to pH 7.2 using 0.5 N NaOH (02153495.5, MP Biomedicals Inc., Pittsburgh, PA), 1X phosphate‐buffered saline (PBS, 10010049 Gibco, Waltham, MA), and 10X PBS (BP2944100, Fisher BioReagants, Pittsburgh, PA).

### 4.2 Rheological Gel Characterization

Collagen solutions were prepared as described above and 150 µL of solution were pipetted into cylindrical rubber gasket mold (8 mm diameter × 3 mm height) and covered with a glass cover slip to ensure a flat surface. The solutions were incubated at room temperature for 5 min before being moved to an incubator at 37°C with 5% CO_2_ to polymerize overnight. The next day the collagen hydrogels were gently removed from the molds and soaked in Endothelial Growth Medium 2 (EGM‐2, NC9525043, Lonza Walkersville CC3162, Basel, Switzerland) supplemented with 10% fetal bovine serum (FB12999102, Fisher Scientific, Pittsburgh, PA) and either 150 ng mL^−1^ of human recombinant lysyl oxidase (LOX, TP313323, OriGene, Rockville, MD) or no LOX for 5 days with daily media changes. LOX stock solutions were stored at 15 µg mL^−1^ in 25 mM Tris HCl (50‐213‐710, Fisher Scientific, Mt. Prospect, IL), 100 mM glycine (G‐630‐1, Gold Biotechnology, St. Louis, MO), and 10% glycerol (A16205, Thermo Fisher, Ward Hill, MA) at pH 7.3. For conditions that did not receive LOX, an equivalent volume of this suspension solution without LOX was added to the media. Oscillatory rheology was executed with a Discovery HR20 rheometer (TA Instruments, New Castle, DE) using an 8‐mm sandblasted Peltier Plate geometry. Collagen gels were compressed to an initial axial force of 0.2 N and oscillatory time sweeps (0.5 rad s^−1^, 2.5% strain) were performed for 3 min to measure the storage and loss moduli.

### 4.3 Confocal Reflectance Microscopy

Collagen solutions were prepared as above and 150 μL was pipetted onto glass bottom dishes (P50G‐1.5‐14‐F, MatTek Corporation, Ashland, MA) and incubated at room temperature for 5 min before being moved to an incubator at 37°C with 5% CO_2_ to polymerize overnight. After polymerization, the hydrogels were covered with EGM‐2 supplemented with 10% FBS and either 150 ng mL^−1^ of LOX or no LOX for 5 days with daily media changes. Pores in the collagen were imaged using confocal reflectance as previously reported [67]. In brief, a Nikon A1R HD25 Laser Scanning Confocal Microscope run by Nikon Elements software and equipped with a 405‐nm laser and a Nikon 20x/0.95 (NA) water immersion objective was used to take the images.

### 4.4 Microfluidic Fabrication

The “infection‐on‐a‐chip” devices were made as previously reported [46, 68]. Briefly, top and bottom masters for the devices we purchased from Protolabs Inc. using their PC‐Like Advanced High Temp (Accura 5530) resin with a natural finish. Polydimethylsiloxane (PDMS, 24236‐10, Electron Microscopy Sciences, Hatfield, PA) was polymerized on the 3D printed masters at 60°C for 24 h. The PDMS layers were then aligned using a light microscope and a PDMS rod (0.337‐mm inner diameter) was inserted between the two layers. The devices were then bonded to a glass‐bottom dish (P50G‐1.5‐30‐F, MatTek Corporation, Ashland, MA) using oxygen plasma from a PE25‐JW Plasma Etcher (Plasma Etch, Carson City, NV).

### 4.5 HUVEC Culture

Pooled human umbilical vein endothelial cells (HUVEC, 50‐305‐964, Promocell GmbH C12203, Heidelberg, Germany) were grown in Endothelial Growth Medium 2 (EGM‐2, NC9525043, Lonza Walkersville CC3162, Basel, Switzerland) to 80% confluency, then passed through passage 6.

### 4.6 *Pseudomonas aeruginosa* Culture

*P. aeruginosa* was prepared as described previously [46]. In brief, *P. aeruginosa* strain K was streaked onto LB plates and incubated for 16 h at 37°C. A single colony was grown in a bacterial shaker at 37°C overnight. The following morning, the culture was diluted in 1:4 in fresh LB broth and grown in a bacteria shaker for 1.5 h at 37°C. 1 mL of bacterial culture was pelleted by centrifugation (17 000 × g for 1 min) and resuspended in 100 μL of EGM‐2. The optical density (OD) was measured, and the bacterial solution was diluted in EGM‐2 to an OD of 5 (measured at 600 nm, 1.25 × 10^6^ CFU mL^−1^). The EGM‐2 contains gentamicin, which prevents replication of the bacteria.

### 4.7 Device and Collagen Preparation

Microfluidic devices were sterilized and prepared as reported previously [46, 47, 48, 68]. The microfluidic devices were sterilized using UV in a biosafety cabinet for 15 min. The central chambers were then incubated for 10 min at room temperature with a 2% polyethylenimine solution in deionized water (03880, Sigma–Aldrich, St. Louis, MO), followed by incubation for 30 min at room temperature with a 0.4% glutaraldehyde solution in deionized water (G6257, Sigma–Aldrich, St. Louis, MO). Collagen was then pipetted into the central device chambers around the PDMS rod and polymerization began for 5 min at room temperature before being placed in an incubator at 37°C with 5% CO_2_. The collagen fully polymerized overnight. The PDMS rods were then removed, leaving a cylindrical void. EGM‐2 supplemented with 10% FBS and either 150 ng mL^−1^ of LOX or no LOX was pipetted through the lumen channel and into the top and bottom ports of the devices once a day for 5 days. HUVECs were then seeded in the lumens at 20 000 cells μL^−1^. The devices were placed in the incubator and flipped every 15 min for 1 h before performing a media change with EGM‐2. Devices were cultured for 2 days, and EGM‐2 media were changed twice daily.

### 4.8 Pore Size Analysis

Images were loaded into Matlab (MathWorks, 2024b). Pixels with a value greater than the median value were assigned a binary value of 0 (fibril), while pixels with a value less than the median image value were set to 1 (pore). The resulting binary images were smoothed by nonlinear filtering (mean value of neighboring pixels from a 3 × 3 kernel), thereby removing noise. Clusters of pixels with the same values were grouped as objects using the bwlabel function in Matlab and the regionprops function was used to calculate, for each pore, the areas and minor axis lengths.

### 4.9 Stained Endothelial Lumens

Endothelial lumens were fixed and stained as has been done previously [46]. Two days after device preparation, 3 μL of *P. aeruginosa* strain K in EGM‐2 was added to the top port of each device and incubated for 2 h before fixing for 30 min with 4% paraformaldehyde (PFA, AAJ19943K2, Thermo Scientific, Waltham, MA) in phosphate‐buffered saline (PBS, B2944‐100, Thermo Scientific, Waltham, MA) at 37°C. Three PBS washes were used to remove the PFA. PBST (PBS with 0.1% Tween 20) was pipetted into the lumens and incubated for 10 min at room temperature. Lumens were incubated overnight at 4°C with 5 μg mL^−1^ Hoescht (23491‐45‐4, Sigma–Aldrich, St. Louis, MO), anti‐ICAM‐1 (1:200, BBA20, R&D systems, Minneapolis, MN), phalloidin (1:200, ab176757, Abcam, Cambridge, United Kingdom), and anti‐VE‐cadherin (1:120, 561567, BD Pharmingen, Franklin Lakes, NJ) in PBST. The next morning, 3 PBS washes were performed to remove unconjugated staining molecules. Single timepoint Z‐stacks were taken every 2 μm along the *Z*‐axis using a Nikon A1R HD25 Laser Scanning Confocal Microscope equipped with a Nikon 20x/0.95 (NA) water immersion objective and run by Nikon Elements software.

### 4.10 Gel Preparation and Cell Culture in Plates

Collagen gels were prepared as described above and 100 μL of solution was pipetted into each well of a 24‐well plate (P24‐1.5H‐N, Cellvis, Mountain View, CA). The collagen solutions were polymerized for 5 min at room temperature before being placed in an incubator at 37°C with 5% CO_2_. The next morning, EGM‐2 supplemented with 10% FBS and 150 ng mL^−1^ LOX or no LOX (Control Condition) was added to each well. This was repeated each day for a total of 5 days of collagen treatment. HUVECs were then seeded 3 × 10^6^ cells/well and cultured for 2 days with daily EGM‐2 media changes.

### 4.11 Staining Endothelial Cells in Plates

*P. aeruginosa* was cultured and prepared as described above, with bacterial solutions being diluted to a final OD of 0.05 in EGM‐2 (measured at 600 nm, 1.25 × 10^4^ CFU mL^−1^). To each well of HUVECs in the 24‐well plate, 100 μL of bacteria solution was added and left to incubate for 2 h. The activated cells were then fixed and stained as described above. The wells were imaged using single timepoint Z‐stacks, with images taken every 5 μm in the *Z*‐axis using a Nikon A1R HD25 Laser Scanning Confocal Microscope equipped with a Nikon 20x/0.95 (NA) water immersion objective run by Nikon Elements software.

### 4.12 Nuclei Count and Stain Intensity Analysis

HUVECs were stained with Hoechst, ICAM‐1, phalloidin, and anti‐VE‐cadherin and imaged as described above in microfluidic devices. A maximum intensity projection along the *Z*‐axis was produced for the bottom half of each device. The number of nuclei in a 550 × 300 μm region of interest placed in the center of each lumen was counted for each replicate. The mean pixel intensities were calculated for the 488 nm channel (ICAM‐1) and 640 nm channel (VE‐cadherin) in the same region of interest for each replicate.

### 4.13 Quantifying Plate Cultured Nuclei Count and Stain Intensities

A maximum intensity projection along the *Z*‐axis was created from the images collected as described above. A region of interest 550 × 300 μm was overlaid in the center of each image. The nuclei count and mean pixel intensities of the 488 nm channel (ICAM‐1) and 640 nm channel (VE‐cadherin) were calculated within this region of interest.

### 4.14 *P. aeruginosa* Dispersal in Infection‐on‐a‐Chip Device

Using devices containing type I collagen treated with control solution or LOX solution for 5 days (as described above), endothelial cells were seeded into the device and cultured for ~2 days. *P. aeruginosa* was cultured overnight (as described above) and 1 mL of that solution was combined with 4 mL fresh media and placed in a shaking rack in an incubator at 37°C for ~45 min. 1 mL of that solution was spun down at 17 000 g for 1 min and then resuspended in 500 μL of 10 nM CellTracker Red CMTPX in 1X PBS and incubated in a water bath for 15 min. Next, cells were spun down at 17 000 g and resuspended in 500 μL 1X PBS. This step was repeated once more with 1X PBS and then again with EGM‐2. Lastly, the cells were resuspended in EGM‐2 to OD = 5.0. 3.0 μL of the bacteria was added to the top port of the device and then placed on the confocal microscope with environmental chamber at 37°C, 5.0% CO2, and 90% relative humidity to image. For each lumen, a 400 μm z‐stack was imaged in 10‐μm intervals every 10 min for 8 h.

### 4.15 Neutrophil Isolation

All blood samples were collected according to the institutional review board‐approved protocols per the Declaration of Helsinki. Peripheral blood neutrophils were isolated from healthy donors, using a MACSxpress Neutrophil Isolation Kit (130‐104‐434, Miltenyi Biotec, Bergisch Gladbach, Germany) and a MACSxpress Erythrocyte Depletion Kit (130‐098‐196, Miltenyi Biotec, Bergisch Gladbach, Germany), according to the manufacturer's instructions. Informed consent was obtained from donors at the time of blood collection according to our institutional review board‐approved protocol number 20‐0082.

### 4.16 Neutrophil Extravasation and Migration

Extravasation and migration experiments were performed as previously reported [46]. Neutrophils were resuspended in PBS at 7.5 million cells mL^−1^ in EGM‐2 after half were stained in 10 nM calcein AM (C3100MP, Thermo Scientific, Waltham, MA). About 5 μL was pipetted into each lumen; then, 3 μL of *P. aeruginosa* in EGM‐2 was added to the top port of each device. The devices were imaged immediately following loading over a Z‐stack of 400 μm with 10 μm steps (for extravasation), then every 30 s for 10 min (for migration) every hour for 8 h.

### 4.17 Integrated Intensity and Diffusive Permeability Analysis

Permeability experiments were run as described previously [46]. Two days after device preparation, 3 μL of either EGM‐2 or *P. aeruginosa* in EGM‐2 was added to the top port of each device; then, devices were incubated for 2 h at 37°C with 5% CO_2_. The media in the lumens was replaced with about 4 μL of 12.5 μM of 10 kDa fluorescein isothiocyanate labeled dextran (FD10S, Sigma) in EGM‐2 and immediately imaged. Images were taken every 5 s for 15 min in the middle Z‐plane of each device. The videos were transferred to FIJI (ImageJ) for analysis. The region of interest for each image consisted of half the total image width (centered along the *x* axis) and a height of three lumen diameters (centered on the lumen along the *y* axis). The integrated intensity was calculated as the sum of the pixel intensities in the top third of the region of interest. Then, a custom MATLAB script, adapted from Polachec et al., was used to calculate the diffusive permeability for each device [52].

### 4.18 Image Acquisition

All imaging was performed on a Nikon A1R HD25 Laser Scanning Confocal Microscope built on the Nikon TI2‐E Inverted Microscope System with a fully automated stage and Nikon Elements acquisition software. Images for confocal reflectance and stained lumens were taken with a Nikon 20x/0.95 (NA) water immersion objective. Extravasation and permeability experiments were imaged with a Nikon 10x/0.45 (NA) objective. Migration experiments were imaged with a Nikon 20x/0.75 (NA) objective. A cage incubator (H201‐T‐Unit‐BL, Oko Labs, Sewickley, PA) was used to perform extravasation, permeability, and migration experiments at 37°C and 5% CO_2_.

### 4.19 Image Processing and Data Analysis

Extravasation and migration experiments were analyzed in the Nikon Elements software. Max intensity projections were created for each extravasation and migration file in the *Z*‐axis. Neutrophils were analyzed in the center of the device to eliminate edge effects. Extravasation analysis was performed as described previously [46]. Briefly, a 690 × 345 μm region of interest was created, defined by the top edge of the lumen in the center of the device. The number of neutrophils was counted within this region at each time point. The number of extravasated neutrophils was normalized to the initial number of neutrophils in the lumen at each timepoint to account for any variability in cell loading. The initial number of neutrophils was determined by drawing a 345 × 122.5 μm rectangular region at the center of the lumen at the first timepoint, and neutrophils in this region were counted.

Extravasated neutrophils that remained in frame for at least half of the timepoints were tracked for migration experiments using the cell motility tracking function in Nikon Elements.

### 4.20 Statistical Analysis

For extravasation, migration, rheology, stained image, and confocal reflectance microscopy experiments, data were pooled from at least three independent replicates. Statistical analysis was performed using Student's *T*‐test to compare the control (CTRL) condition to the lysyl oxidase (LOX) condition with an alpha value of 0.05 followed by a Tukey‐Kramer adjustment, unless otherwise noted. Graphs show mean values ± standard error of the mean (SEM). *p*‐values are labeled as *p < 0.05, and **p < 0.01, unless otherwise noted.

## Supporting Information

Additional supporting information can be found online in the Supporting Information section. **Supporting Fig. S1:** **Storage**. **(A)** Storage (G′) and **(B)** Loss (G″) moduli of LOX‐crosslinked (LOX) and uncrosslinked (Ctrl) hydrogels, measured at 10 rad s^−1^, 1.0% strain measured at 37 °C. Error bars indicate mean ± SEM of 4 gels per condition per timepoint. **(C)** Table of P‐values comparing Storage and Loss moduli of Ctrl gels to LOX gels after 1, 3, and 5 days of treatment. Independent samples t‐tests were performed for each modulus and day of treatment, with a Bonferroni correction to account for multiple comparisons with an adjusted alpha value of 0.0083. **Supporting Fig. S2:** **Pore distributions of lysyl oxidase and untreated collagen gels**. Histograms showing distributions of pores in LOX‐crosslinked (LOX) and uncrosslinked (Ctrl) collagen gels, with respect to **(A)** area and **(B)** M.A.L. For A, bins show the number of pores with areas between 0.75 and 5 μm^2^ with bin widths of 0.25 μm^2^. For B, bins show the number of pores with M.A.L. between 0.5 and 10 μm with bin widths of 0.25 μm. **Supporting Fig. S3:** **The addition of LOX to device does not affect *P. aeruginosa* migration**. Representative images of *P. aeruginosa* added to the top port of the device as it distributes through the device over the course of 8 hours. Cells were stained with CellTracker Red CMTPX. **(A)** Max intensity projections of the bacteria as it distributes in the device (scale bar = 250 μm). **(B)** 3D projections of the bacteria created in ImageJ to show bacterial distribution in the z‐plane over 400 μm (scale bar = 250 μm). **Supporting Fig. S4:** **Neutrophil migration length and straightness in lysyl oxidase or untreated collagen matrices**. Migration properties, **(A)** path length and **(B)** straightness, of neutrophils measured over 10‐minute increments every hour for 8 hours post stimulation with *P. aeruginosa*. Extravasated neutrophils, from 20 (uncrosslinked, Ctrl) and 19 (LOXcrosslinked, LOX) devices across 4 independent experiments and 4 neutrophil donors, were tracked the cell motility function in Nikon's Elements software. Ctrl and LOX conditions were compared to each other at each time point using Student's t‐test with an alpha value of 0.05. Error bars indicate the means ± SEM. **Supporting Fig. S5:** **Lysyl oxidase crosslinking of collagen in well plates**. **(A)** Representative maximum intensity projections of confocal images of HUVECs seeded in 96‐well plates on top of LOX‐crosslinked (LOX) or uncrosslinked (Ctrl) collagen gels and stained with Hoechst (nuclei, blue), anti‐ICAM‐1 (adhesion molecules, green), phalloidin (actin, red), anti‐VEcadherin (junctions, far red) 2 hours post stimulation with *P. aeruginosa* (scale bar = 250 μm). Bar graphs of **(B)** the number of nuclei counted, **(C)** mean pixel intensity of ICAM‐1, and **(D)** mean pixel intensity of VE‐cadherin in a fixed region of interest about the bottom of each lumen. Data is shown for 3 wells per condition across 1 independent experiments.

## Funding

This work was supported by the National Science Foundation Graduate Research Fellowship under Grant No. DGE 2040434.

## Ethics Statement

The studies involving humans were approved by University of Colorado Institutional Review Board, protocol number 20‐0082. The studies were conducted in accordance with the local legislation and institutional requirements. The participants provided their written informed consent to participate in this study.

## Conflicts of Interest

The authors declare no conflicts of interest.

## Supporting information

Supplementary Material

## Data Availability

The raw data supporting the conclusions of this article will be made available by the authors, without undue reservation.

## References

[1] R. Grecian , M. K. B. Whyte , and S. R. Walmsley , ”The Role of Neutrophils in Cancer,“ British Medical Bulletin 128 (2018): 5–14. 30137312 10.1093/bmb/ldy029 PMC6289220

[2] J. D. Puerta‐arias , P. A. Pino‐tamayo , J. C. Arango , and A. Gonzalez , “Depletion of Neutrophils Promotes the Resolution of Pulmonary Inflammation and Fibrosis in Mice Infected with Paracoccidioides Brasiliensis,” PLoS One 11 (2016): e0163985–23. 27690127 10.1371/journal.pone.0163985 PMC5045199

[3] G. Wang and W. M. Nauseef , “Neutrophil Dysfunction in the Pathogenesis of Cystic Fibrosis,” Blood 139 (2022): 2622–2631. 35213685 10.1182/blood.2021014699 PMC9053701

[4] B. L. Rapoport , H. C. Steel , A. J. Theron , T. Smit , and R. Anderson , “Role of the Neutrophil in the Pathogenesis of Advanced Cancer and Impaired Responsiveness to Therapy,” Molecules 25 (2020): 1–22. 10.3390/molecules25071618 PMC7180559 32244751

[5] O. Soehnlein , “Multiple Roles for Neutrophils in Atherosclerosis,” Circulation Research 110 (2012): 875–888. 22427325 10.1161/CIRCRESAHA.111.257535

[6] T. Németh , M. Sperandio , and A. Mócsai , “Neutrophils as Emerging Therapeutic Targets,” Nature Reviews Drug Discovery 19 (2020): 253–275. 31969717 10.1038/s41573-019-0054-z

[7] Y.‐T. Gong , L.‐J. Zhang , Y.‐C. Liu , et al., “Neutrophils as Potential Therapeutic Targets for Breast Cancer,” Pharmacological Research 198 (2023): 1–17. 10.1016/j.phrs.2023.106996 37972723

[8] I. Salken , J. J. Provencio , and A. P. Coulibaly , A Potential Therapeutic Target: The Role of Neutrophils in the Central Nervous System,“ Brain, Behavior, & Immunity ‐ Health 33 (2023): 1–8. 10.1016/j.bbih.2023.100688 PMC10520304 37767236

[9] M. Bartneck and J. Wang , ”Therapeutic Targeting of Neutrophil Granulocytes in Inflammatory Liver Disease,“ Frontiers in Immunology 10 (2019): 2257. 31616430 10.3389/fimmu.2019.02257 PMC6764082

[10] G. Hajishengallis and T. Chavakis , “Mechanisms and Therapeutic Modulation of Neutrophil‐Mediated,” Inflammation Journal of Dental Research 101 (2022): 1563–1571. 35786033 10.1177/00220345221107602 PMC9703529

[11] Z. G. Guvatova , P. V. Borisov , A. A. Alekseev , and A. A. Moskalev , ”Age‐Related Changes in Extracellular Matrix,“ Biochemistry 87 (2022): 1535–1551. 36717445 10.1134/S0006297922120112

[12] V. Mohan , A. Das , and I. Sagi , “Emerging Roles of ECM Remodeling Processes in Cancer,” Seminars in Cancer Biology 62 (2020): 192–200. 31518697 10.1016/j.semcancer.2019.09.004

[13] T. N. Wight and S. Potter‐Perigo , “The Extracellular Matrix: An Active or Passive Player in Fibrosis?,” American Journal of Physiology‐Gastrointestinal and Liver Physiology 301 (2011): G950–G955. 21512158 10.1152/ajpgi.00132.2011 PMC3233785

[14] R. B. Diller and A. J. Tabor , “The Role of the Extracellular Matrix (ECM) in Wound Healing: A Review,” Biomimetics 7 (2022): 14–16. 35892357 10.3390/biomimetics7030087 PMC9326521

[15] N. Yang , D. F. Cao , X. X. Yin , H. H. Zhou , and X. Y. Mao , “Lysyl Oxidases: Emerging Biomarkers and Therapeutic Targets for Various Diseases,” Biomedicine and Pharmacotherapy 131 (2020): 110791. 33152948 10.1016/j.biopha.2020.110791

[16] Y. F. I. Setargew , K. Wyllie , R. D. Grant , J. L. Chitty , and T. R. Cox , “Targeting Lysyl Oxidase Family Meditated Matrix Cross‐Linking as an Anti‐Stromal Therapy in Solid Tumours,” Cancers 13 (2021): 1–26. 10.3390/cancers13030491 PMC7865543 33513979

[17] A. M. Baker , D. Bird , G. Lang , T. R. Cox , and J. T. Erler ,“ Lysyl Oxidase Enzymatic Function Increases Stiffness to Drive Colorectal Cancer Progression through FAK,” Oncogene 32 (2013): 1863–1868. 22641216 10.1038/onc.2012.202

[18] J. Martínez‐González , S. Varona , L. Cañes , et al., “Emerging Roles of Lysyl Oxidases in the Cardiovascular System: New Concepts and Therapeutic Challenges,” Biomolecules 9 (2019): 610. 31615160 10.3390/biom9100610 PMC6843517

[19] F. Rodriguez‐Pascual and T. Rosell‐Garcia , “Lysyl Oxidases: Functions and Disorders,” Journal of Glaucoma 27 (2018): S15–S19. 29419646 10.1097/IJG.0000000000000910

[20] P. W. Oakes , D. C. Patel , N. A. Morin , et al., “Neutrophil Morphology and Migration Are Affected by Substrate Elasticity,” Blood 114 (2009): 1387–1395. 19491394 10.1182/blood-2008-11-191445 PMC2727411

[21] K. M. Stroka and H. Aranda‐Espinoza , “Neutrophils Display Biphasic Relationship between Migration and Substrate Stiffness,” Cell Motility and the Cytoskeleton 66 (2009): 328–341. 19373775 10.1002/cm.20363

[22] K. M. Stroka , H. N. Hayenga , H. Aranda‐Espinoza , and H. Mellor , “Human Neutrophil Cytoskeletal Dynamics and Contractility Actively Contribute to Trans‐Endothelial Migration,” PLoS ONE 8 (2013): e61377. 23626676 10.1371/journal.pone.0061377 PMC3634075

[23] B. Hamza , E. Wong, S. Patel, H. Cho, J. Martel, and D. Irimia, Retrotaxis of Human Neutrophils during Mechanical Confinement inside Microfluidic Channels.,“ Integrative Biology 6 (2014): 175–183. 24419464 10.1039/c3ib40175h PMC3928968

[24] X. Wang , E. Jodoin , J. Jorgensen , et al., “Progressive Mechanical Confinement of Chemotactic Neutrophils Induces Arrest, Oscillations, and Retrotaxis,” Journal of Leukocyte Biology 104 (2018): 1253–1261. 30129679 10.1002/JLB.5TA0318-110RRR PMC6258301

[25] K. M. Stroka and H. Aranda‐Espinoza , “Endothelial Cell Substrate Stiffness Influences Neutrophil Transmigration via Myosin Light Chain Kinase‐Dependent Cell Contraction,” Blood 118 (2011): 1632–1640. 21652678 10.1182/blood-2010-11-321125 PMC3156049

[26] R. A. Jannat , G. P. Robbins , B. G. Ricart , M. Dembo , and D. A. Hammer , “Neutrophil Adhesion and Chemotaxis Depend on Substrate Mechanics,” Journal of Physics Condensed Matter 22 (2010): 1–14. 10.1088/0953-8984/22/19/194117 PMC2867619 20473350

[27] L. Erpenbeck , A. L. Gruhn , G. Kudryasheva , et al.,” Effect of Adhesion and Substrate Elasticity on Neutrophil Extracellular Trap Formation,“ Frontiers in Immunology 10 (2019): 2320. 31632402 10.3389/fimmu.2019.02320 PMC6781793

[28] S. J. Henry , C. S. Chen , J. C. Crocker , and D. A. Hammer , “Protrusive and Contractile Forces of Spreading Human Neutrophils,” Biophysical Journal 109 (2015): 699–709. 26287622 10.1016/j.bpj.2015.05.041 PMC4547143

[29] A. Schaefer , J. te Riet , K. Ritz , et al., “Actin‐Binding Proteins Differentially Regulate Endothelial Cell Stiffness, ICAM‐1 Function and Neutrophil Transmigration,” Journal of Cell Science 127 (2014): 4470–4985. 25107367 10.1242/jcs.154708

[30] T. Jiang , X.‐Y. Tang , Y. Mao , et al., “Matrix Mechanics Regulate the Polarization State of Bone Marrow‐Derived Neutrophils through the JAK1/STAT3 Signaling Pathway,” Acta Biomaterialia 168 (2023): 159–173. 37467837 10.1016/j.actbio.2023.07.012

[31] D. Vestweber , “How Leukocytes Cross the Vascular Endothelium,” Nature Reviews Immunology 15 (2015): 692–704. 10.1038/nri3908 26471775

[32] M. D. Filippi , “Neutrophil Transendothelial Migration: Updates and New Perspectives,” Blood 133 (2019): 2149–2158. 30898863 10.1182/blood-2018-12-844605 PMC6524565

[33] W. Chen , B. Tian , J. Liang , S. Yu , Y. Zhou , and S. Li , “Matrix Stiffness Regulates the Interactions between Endothelial Cells and Monocytes,” Biomaterials 221 (2019): 119362. 31442696 10.1016/j.biomaterials.2019.119362

[34] E. E. Bastounis , Y. T. Yeh , and J. A. Theriot ,” Matrix Stiffness Modulates Infection of Endothelial Cells by Listeria Monocytogenes via Expression of Cell Surface Vimentin,“ Molecular Biology of the Cell 29 (2018): 1571–1589. 29718765 10.1091/mbc.E18-04-0228 PMC6080647

[35] F. J. Byfield , R. K. Reen , T. P. Shentu , I. Levitan , and K. J. Gooch , “Endothelial Actin and Cell Stiffness Is Modulated by Substrate Stiffness in 2D and 3D,” Journal of Biomechanical Engineering 42 (2009): 1114–1119. 10.1016/j.jbiomech.2009.02.012 PMC2893018 19356760

[36] L. D. Santos , G. Fuhrmann , M. Juenet , et al., “Extracellular Stiffness Modulates the Expression of Functional Proteins and Growth Factors in Endothelial Cells,” Advanced Healthcare Materials 4 (2015): 2056–2063. 26270789 10.1002/adhm.201500338

[37] J. C. Kohn , D. W. Zhou , F. Bordeleau , et al., “Cooperative Effects of Matrix Stiffness and Fluid Shear Stress on Endothelial Cell Behavior,” Biophysical Journal 108 (2015): 471–478. 25650915 10.1016/j.bpj.2014.12.023 PMC4317546

[38] H. Jeon , J. H. Tsui , S. I. Jang , et al., “Combined Effects of Substrate Topography and Stiffness on Endothelial Cytokine and Chemokine Secretion,” ACS Applied Materials & Interfaces 7 (2015): 4525–4532. 25658848 10.1021/acsami.5b00554 PMC4937831

[39] J. Huynh , N. Nishimura , K. Rana , et al., “Age‐Related Intimal Stiffening Enhances Endothelial Permeability and Leukocyte Transmigration,” Science Translational Medicine 3 (2011): 1–9. 10.1126/scitranslmed.3002761 PMC3693751 22158860

[40] C. Rodriguez , J. Martinez‐Gonzalez , B. Raposo , J. F. Alcudia , A. Guadall , and L. Badimon , “Regulation of Lysyl Oxidase in Vascular Cells: Lysyl Oxidase as a New Player in Cardiovascular Diseases,” Cardiovascular Research 79 (2008): 7–13. 18469024 10.1093/cvr/cvn102

[41] T. Osawa , N. Ohga , K. Akiyama , et al., “Lysyl Oxidase Secreted by Tumour Endothelial Cells Promotes Angiogenesis and Metastasis,” British Journal of Cancer 109 (2013): 2237–2247. 24045659 10.1038/bjc.2013.535 PMC3798951

[42] A.‐M. Baker , D. Bird , J. C. Welti , et al., “Lysyl Oxidase Plays a Critical Role in Endothelial Cell Stimulation to Drive Tumor Angiogenesis,” Cancer Research 73 (2013): 583–594. 23188504 10.1158/0008-5472.CAN-12-2447 PMC3548904

[43] J. Reibman , S. Meixler , T. C. Lee , et al., “Transforming Growth Factor β1, a Potent Chemoattractant for Human Neutrophils, Bypasses Classic Signal‐Transduction Pathways,” Proceedings of the National Academy of Sciences 88 (1991): 6805–6809. 10.1073/pnas.88.15.6805 PMC52177 1650483

[44] P. Atsawasuwan , Y. Mochida , M. Katafuchi , et al., “Lysyl Oxidase Binds Transforming Growth Factor‐β and Regulates Its Signaling via Amine Oxidase Activity,” Journal of Biological Chemistry 283 (2008): 34229–34240. 18835815 10.1074/jbc.M803142200 PMC2590693

[45] R. Laczko and K. Csiszar , “Lysyl Oxidase (Lox): Functional Contributions to Signaling Pathways,” Biomolecules 10 (2020): 1–16. 10.3390/biom10081093 PMC7465975 32708046

[46] C. J. Calo , T. Patil , M. Palizzi , N. Wheeler , and L. E. Hind , “Collagen Concentration Regulates Neutrophil Extravasation and Migration in Response to Infection in an Endothelium Dependent Manner,” Frontiers in Immunology 15 (2024): 1405364. 39021568 10.3389/fimmu.2024.1405364 PMC11251947

[47] I. M. Richardson , C. J. Calo , E. L. Ginter ,et al., “Diverse Bacteria Elicit Distinct Neutrophil Responses in a Physiologically Relevant Model of Infection,” iScience 27 (2024): 1–17. 10.1016/j.isci.2023.108627 PMC10770534 38188520

[48] L. E. Hind , P. N. Ingram , D. J. Beebe , and A. Huttenlocher , “Interaction with an Endothelial Lumen Increases Neutrophil Lifetime and Motility in Response to P Aeruginosa,” Blood 132 (2018): 1818–1828. 30143504 10.1182/blood-2018-05-848465 PMC6202912

[49] T. R. Cox , D. Bird , A.‐M. Baker , et al., “LOX‐Mediated Collagen Crosslinking Is Responsible for Fibrosis‐Enhanced Metastasis,” Cancer Research 73 (2013): 1721–1732. 23345161 10.1158/0008-5472.CAN-12-2233 PMC3672851

[50] A. Mammoto , T. Mammoto , M. Kanapathipillai , et al., “Control of Lung Vascular Permeability and Endotoxin‐Induced Pulmonary Oedema by Changes in Extracellular Matrix Mechanics,” Nature Communication 4 (2013): 1759. 10.1038/ncomms2774 23612300

[51] C. Rodríguez , B. Raposo , J. Martínez‐gonzález , L. Casaní , and L. Badimon ,“ Low Density Lipoproteins Downregulate Lysyl Oxidase in Vascular Ednothelial Cells and the Arterial Wall,” Arteriosclerosis, Thrombosis, and Vascular Biology 22 (2002): 1409–1414. 12231558 10.1161/01.atv.0000033818.21748.99

[52] W. J. Polacheck , M. L. Kutys , J. B. Tefft , and C. S. Chen , “Microfabricated Blood Vessels for Modeling the Vascular Transport Barrier,” Nature Protocols 14 (2019): 1425–1454. 30953042 10.1038/s41596-019-0144-8 PMC7046311

[53] B. Hermant , S. Bibert , E. Concord , et al., “Identification of Proteases Involved in the Proteolysis of Vascular Endothelium Cadherin during Neutrophil Transmigration,” Journal of Biological Chemistry 278 (2003): 14002–14012. 12584200 10.1074/jbc.M300351200

[54] L. Yang , R. M. Froio , T. E. Sciuto , A. M. Dvorak , R. Alon , and F. W. Luscinskas ,“ ICAM‐1 Regulates Neutrophil Adhesion and Transcellular Migration of TNF‐Alpha‐Activated Vascular Endothelium under Flow,” Blood 6 (2012): 1992–2011. 10.1182/blood-2004-12-4942 PMC1635241 15811956

[55] S. Chandrakumar , I. Santiago Tierno , M. Agarwal , N. Matisioudis , T. S. Kern , and K. Ghosh , “Subendothelial Matrix Stiffening by Lysyl Oxidase Enhances RAGE‐Mediated Retinal Endothelial Activation in Diabetes,” Diabetes 72 (2023): 973–985. 37058096 10.2337/db22-0761 PMC10281239

[56] S. Tauzin , T. W. Starnes , F. B. Becker , P. Lam , and A. Huttenlocher , “Redox and Src Family Kinase Signaling Control Leukocyte Wound Attraction and Neutrophil Reverse Migration,” Journal of Cell Biology 207 (2014): 589–598. 25488917 10.1083/jcb.201408090 PMC4259815

[57] M. Siwicki and P. Kubes , “Neutrophils in Host Defense, Healing, and Hypersensitivity: Dynamic Cells Within a Dynamic Host,” Journal of Allergy and Clinical Immunology 151 (2023): 634–655. 36642653 10.1016/j.jaci.2022.12.004

[58] C. Turkay , R. Saba , N. Pahin , et al., “Effect of Chronic Pseudomonas Aeruginosa Infection on the Development of Atherosclerosis in a Rat Model,” Clinical Microbiology and Infection 10 (2004): 705–708. 15301672 10.1111/j.1469-0691.2004.00920.x

[59] P. Paprocka , B. Durnas , A. Mankowska , G. Król , T. Wollny , and R. Bucki , ”Pseudomonas aeruginosa Infections in Cancer Patients,“ Pathogens 11 (2022): 1–17. 10.3390/pathogens11060679 PMC9230571 35745533

[60] J. Y. Zhang , W.‐W. Zhu , M.‐Y. Wang , et al., “Cancer‐Associated Fibroblasts Promote Oral Squamous Cell Carcinoma Progression through LOX‐Mediated Matrix Stiffness,” International Journal of Translational Medicine 19 (2021): 1–16. 10.1186/s12967-021-03181-x PMC8686394 34930321

[61] J. Song , X. Zhang , K. Buscher , et al., “Endothelial Basement Membrane Laminin 511 Contributes to Endothelial Junctional Tightness and Thereby Inhibits Leukocyte Transmigration,” Cell Report 18 (2017): 1256–1269. 10.1016/j.celrep.2016.12.092 28147279

[62] F. Bordeleau , B. N. Mason , E. M. Lollis , et al., “Matrix Stiffening Promotes a Tumor Vasculature Phenotype,” Proceedings of the National Academy of Sciences 114 (2017): 492–497. 10.1073/pnas.1613855114 PMC5255592 28034921

[63] M. Drechsler , R. T. A. Megens , M. Van Zandvoort , C. Weber , and O. Soehnlein , “Hyperlipidemia‐Triggered Neutrophilia Promotes Early Atherosclerosis,” Vascular Medicine 122 (2010): 1837–1845. 10.1161/CIRCULATIONAHA.110.961714 20956207

[64] M. Mihlan , K. M. Glaser , M. W. Epple , and T. Lämmermann , “Amoeboid Migration and Swarming Dynamics in Tissues,” Frontiers in Cell and Developmental Biology 10 (2022): 871789. 35478973 10.3389/fcell.2022.871789 PMC9038224

[65] L. E. Hind , W. J. B. Vincent , and A. Huttenlocher ,“ Leading from the Back: The Role of the Uropod in Neutrophil Polarization and Migration,” Developmental Cell 38 (2016): 161–169. 27459068 10.1016/j.devcel.2016.06.031 PMC4982870

[66] K. Wolf , et al., “Physical Limits of Cell Migration: Control by ECM Space and Nuclear Deformation and Tuning by Proteolysis and Traction Force,” Journal of Cell Biology 201 (2013): 1069–1084. 23798731 10.1083/jcb.201210152 PMC3691458

[67] P. V. Taufalele , J. A. VanderBurgh , A. Muñoz , M. R. Zanotelli , and C. A. Reinhart‐King , “Fiber Alignment Drives Changes in Architectural and Mechanical Features in Collagen Matrices,” PLoS One 14 (2019): e0216537. 31091287 10.1371/journal.pone.0216537 PMC6519824

[68] J. A. Jimenez‐Torres , S. L. Peery , K. E. Sung , and D. Beebe , “LumeNEXT: A Practical Method to Pattern Luminal Structures in ECM Gels,” Physiology & Behavior 176 (2017): 139–148. 26610188 10.1002/adhm.201500608 PMC4776323

