# Supplementary material for: Lysyl Oxidase Reduces Neutrophil Extravasation in Response to P. aeruginosa in an Infection‐on‐a‐Chip Model
Source: Adv Nanobiomed Res. 2026 Mar 7;6(7):e202500180. doi: 10.1002/anbr.202500180 (PMC13293553; doi:10.1002/anbr.202500180)
Supplement: Supplementary file 1 — Supplementary Material [file ANBR-6-e202500180-s001.pdf]

Supporting Information

Supporting Information for Lysyl Oxidase Reduces Neutrophil Extravasation in Response to *P. aeruginosa* in an Infection-on-a-Chip Model

*Christopher J. Calo, Tanvi Patil, Josephine Fouts, Eric L. Ginter, M. Radke, H. K. Weppner, J. Owens, Laurel E. Hind**

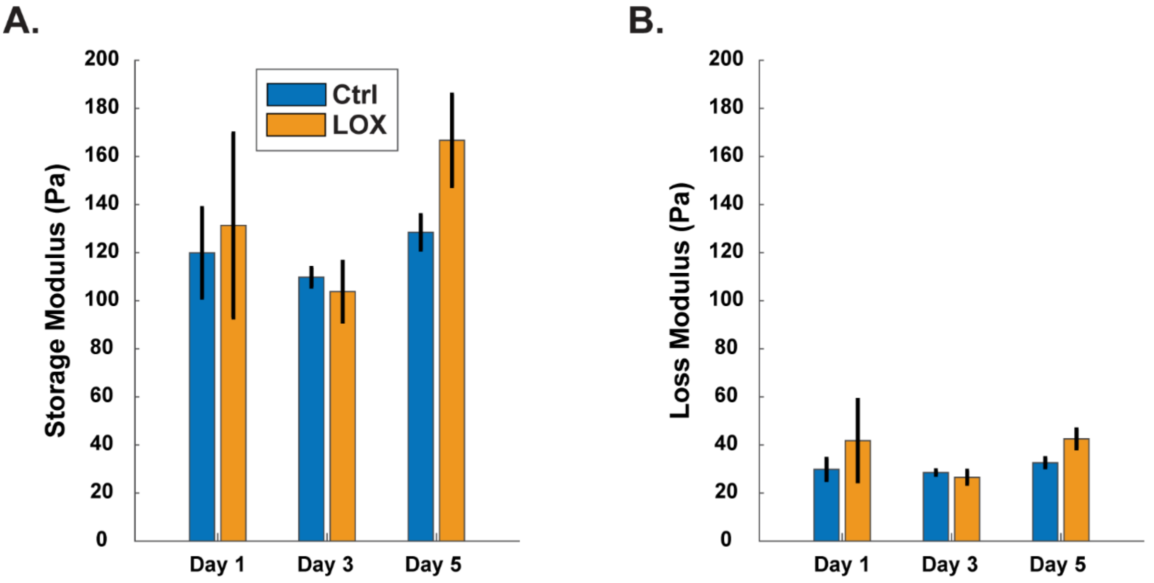

C.

Treatment Time	P-value for Storage Moduli	P-value for Loss Moduli
**Day 1**	P=0.3988	P=0.2599
**Day 3**	P=0.3306	P=0.2601
**Day 5**	P=0.0547	P=0.3312

Figure S1: Storage . (A) Storage (G') and **(B)** Loss (G'') moduli of LOX-crosslinked (LOX) and uncrosslinked (Ctrl) hydrogels, measured at 10 rad s^{-1}, 1.0% strain measured at 37 ^{o}C. Error bars indicate mean ± SEM of 4 gels per condition per timepoint. **(C)** Table of P-values comparing Storage and Loss moduli of Ctrl gels to LOX gels after 1, 3, and 5 days of treatment. Independent samples t-tests were performed for each modulus and day of treatment, with a Bonferroni correction to account for multiple comparisons with an adjusted alpha value of 0.0083.

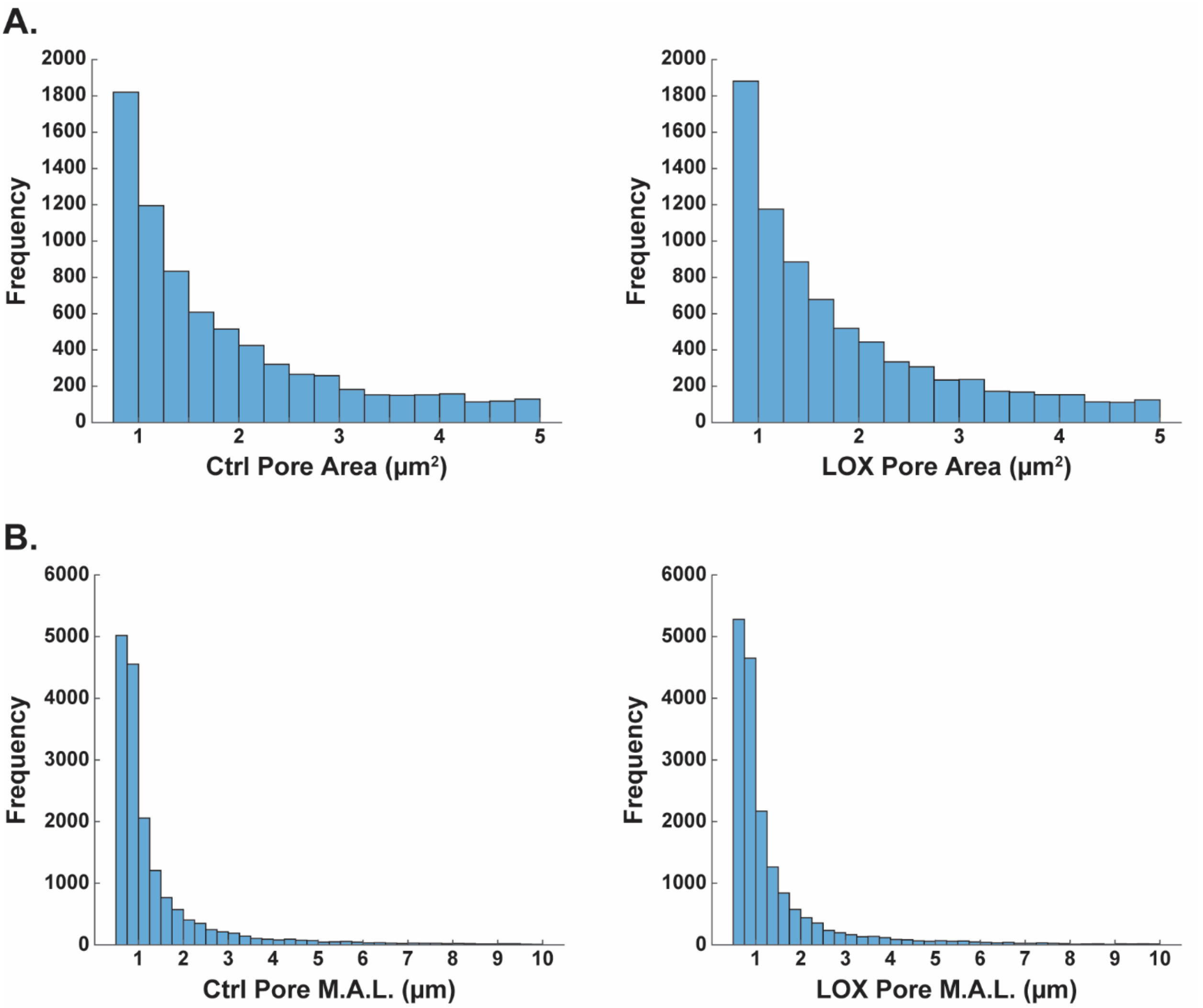

Figure S2: Pore distributions of lysyl oxidase and untreated collagen gels. Histograms showing distributions of pores in LOX-crosslinked (LOX) and uncrosslinked (Ctrl) collagen gels, with respect to **(A)** area and **(B)** M.A.L. For A, bins show the number of pores with areas between 0.75 and 5 μm^2 with bin widths of 0.25 μm^2. For B, bins show the number of pores with M.A.L. between 0.5 and 10 μm with bin widths of 0.25 μm.

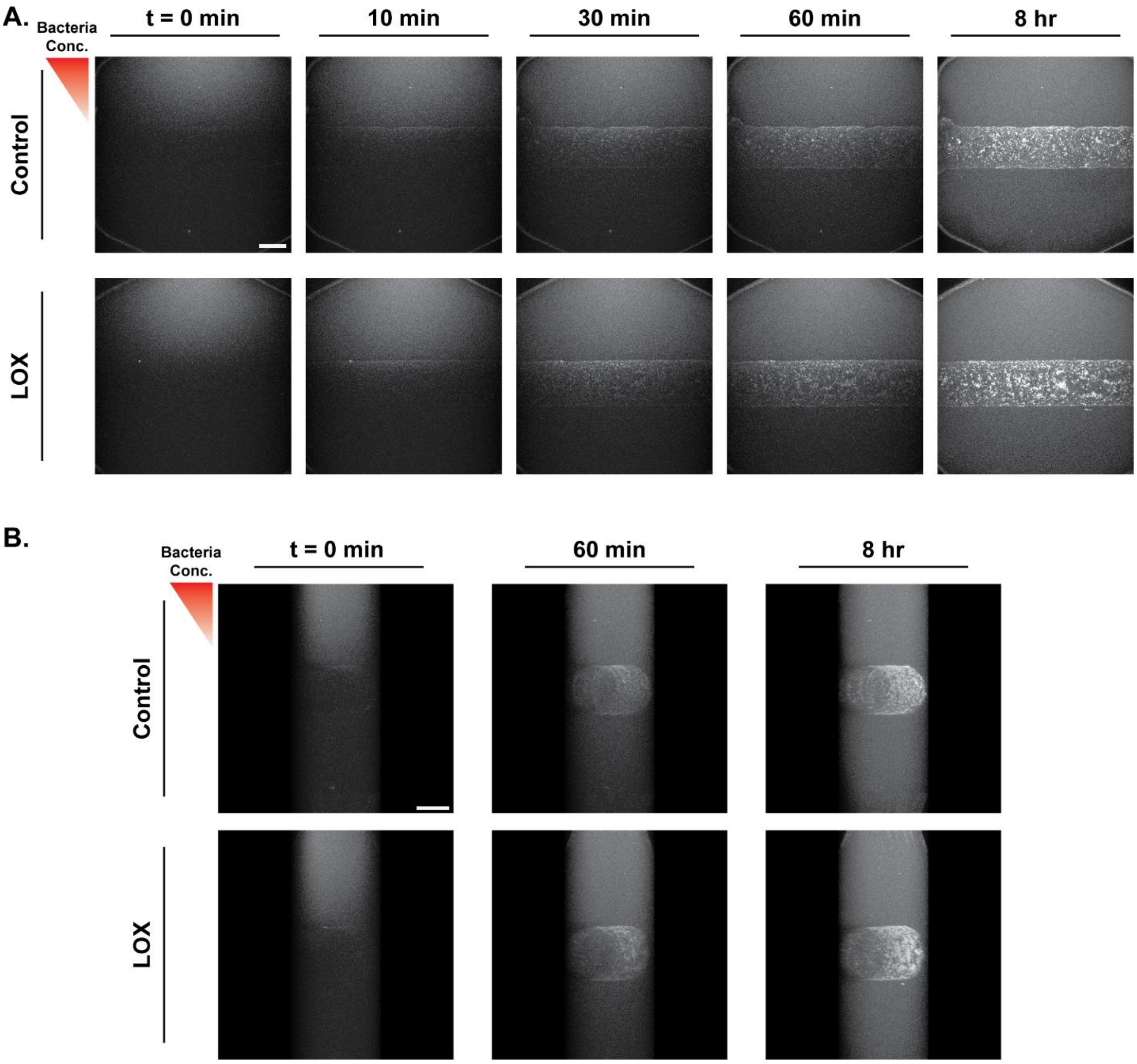

Figure S3: The addition of LOX to device does not affect *P. aeruginosa* migration. Representative images of *P. aeruginosa* added to the top port of the device as it distributes through the device over the course of 8 hours. Cells were stained with CellTracker Red CMTPX. (A) Max intensity projections of the bacteria as it distributes in the device (scale bar = 250 μm). (B) 3D projections of the bacteria created in ImageJ to show bacterial distribution in the z-plane over 400 μm (scale bar = 250 μm).

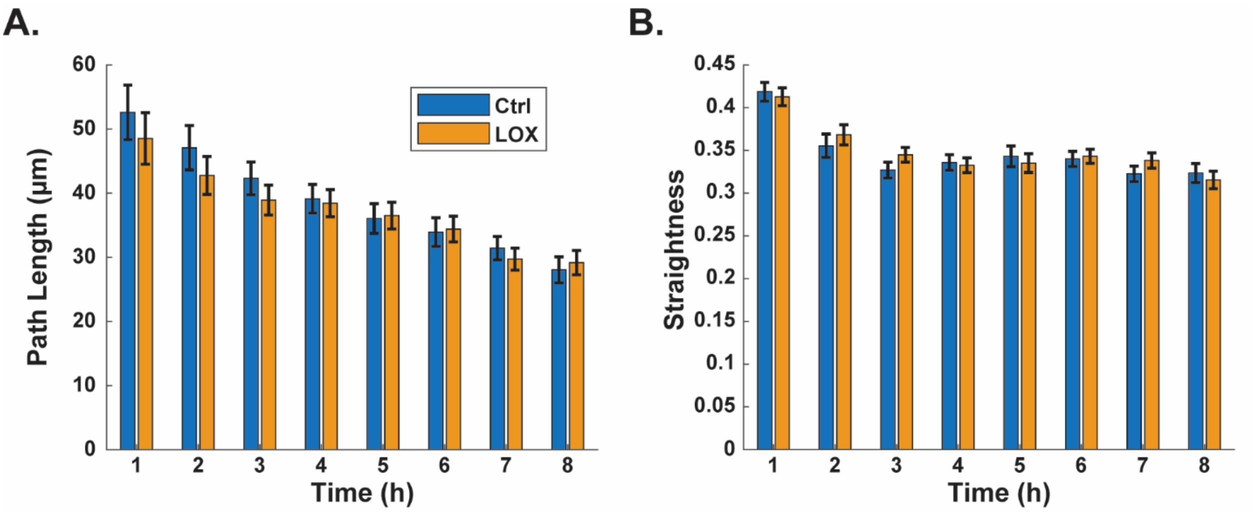

Figure S4: Neutrophil migration length and straightness in lysyl oxidase or untreated collagen matrices. Migration properties, **(A)** path length and **(B)** straightness, of neutrophils measured over 10-minute increments every hour for 8 hours post stimulation with *P. aeruginosa*. Extravasated neutrophils, from 20 (uncrosslinked, Ctrl) and 19 (LOX-crosslinked, LOX) devices across 4 independent experiments and 4 neutrophil donors, were tracked the cell motility function in Nikon's Elements software. Ctrl and LOX conditions were compared to each other at each time point using Student's t-test with an alpha value of 0.05. Error bars indicate the means ± SEM.

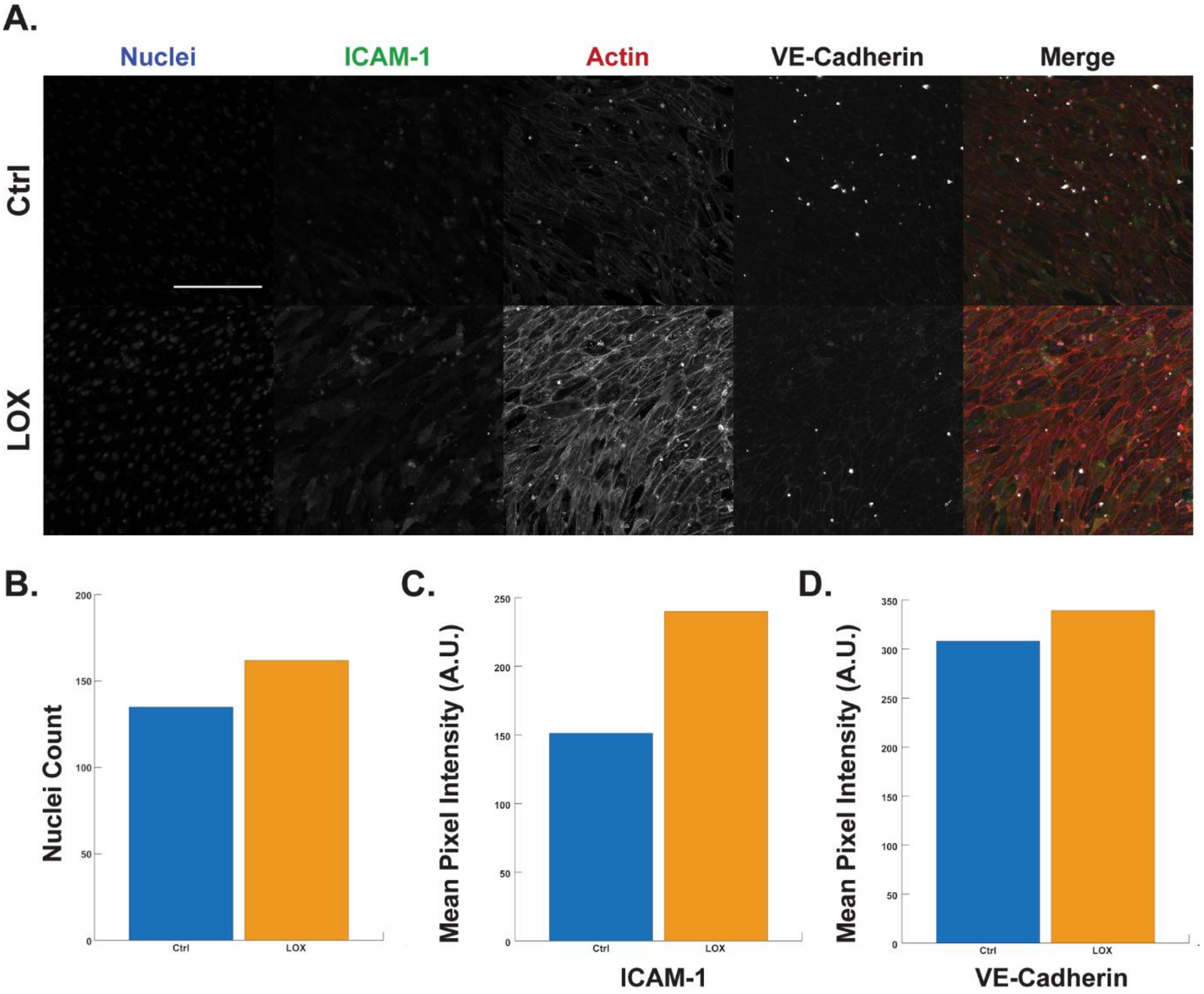

Figure S5. Lysyl oxidase crosslinking of collagen in well plates. (A) Representative maximum intensity projections of confocal images of HUVECs seeded in 96-well plates on top of LOX-crosslinked (LOX) or uncrosslinked (Ctrl) collagen gels and stained with Hoechst (nuclei, blue), anti-ICAM-1 (adhesion molecules, green), phalloidin (actin, red), anti-VE-cadherin (junctions, far red) 2 hours post stimulation with *P. aeruginosa* (scale bar = 250 µm). Bar graphs of **(B)** the number of nuclei counted, **(C)** mean pixel intensity of ICAM-1, and **(D)** mean pixel intensity of VE-cadherin in a fixed region of interest about the bottom of each lumen. Data is shown for 3 wells per condition across 1 independent experiments.
